# Collagenous gastritis, hyperplastic polyp, and intraepithelial duodenal lymphocytosis in a 15-year-old child: An iconographic case report

**DOI:** 10.1097/MD.0000000000045993

**Published:** 2025-11-14

**Authors:** Consolato Sergi, Janice Patry, Mohit Kehar

**Affiliations:** a Department of Pathology and Laboratory Medicine, Children’s Hospital of Eastern Ontario, University of Ottawa, Ottawa, ON, Canada; b Pediatric Gastroenterology, Children’s Hospital of Eastern Ontario, University of Ottawa, Ottawa, ON, Canada.

**Keywords:** case report, collagenous gastritis, electron microscopy, hyperplastic polyp, interleukin-4, intraepithelial duodenal lymphocytosis, villous blunting

## Abstract

**Rationale:**

Collagenous gastritis is an uncommon condition initially identified in 1989 by Colletti and Trainer, characterized by a subepithelial collagen band exceeding 10 μm in thickness. Two patient subgroups have been identified: children and young adults exhibiting severe anemia with illness confined to the gastric mucosa and no colonic involvement, and older individuals presenting with watery diarrhea and collagenous gastritis linked to collagenous colitis. A combination of hyperplastic polyps and Marsh I duodenal lymphocytosis has not been fully reported yet.

**Patient concerns:**

A 15-year-old boy exhibited a prolonged history of fatigue, lethargy, weight reduction, and poor development. He exhibited iron deficiency anemia. Previous oral iron supplementation had been ineffective, necessitating the use of intravenous iron infusions. An endoscopy revealed a nodular, polypoid stomach with small polyps located in the antrum.

**Diagnoses:**

Biopsies revealed characteristics of collagenous gastritis, characterized by a thickened subepithelial band. A polyp exhibited elongated, architecturally distorted pits with outpouchings, cystic dilation, and papillary formation but without dysplasia. Intraepithelial lymphocytes exceeding 30 per 100 epithelial cells were seen in the small intestine. In our patient, pericryptal fibroblasts, inflammatory cells, and a distinctive subepithelial band made of randomly oriented collagen fibers were shown histologically and ultrastructurally.

**Interventions:**

The patient was treated with lansoprazole 30 mg twice daily and famotidine 40 mg once daily, as well as oral iron supplementation.

**Outcomes:**

Clinical improvement was noted, and hemoglobin and ferritin levels were 123 g/L and 9 µg/L, respectively, at 12 months following therapy.

**Lessons:**

In consideration of a colon-rectal cancer diagnosis of his father at the age of 45 years, we carried out a Search Tool for the Retrieval of Interacting Genes/Proteins and Pymol-based Protein Data Bank study and found that interleukin-4 may be the key factor supporting the occurrence of hyperplastic polyps in collagenous gastritis.

## 1. Introduction

The formation of a subepithelial collagen band in the mucosa is a hallmark of both the exceedingly rare collagenous gastritis and the more prevalent collagenous colitis, with fewer than 300 patients reported in the English-language literature. Of these, about one-third have been described as pediatric collagenous gastritis.^[1–22]^

In childhood, collagenous gastritis is very rare, causing severe iron deficiency anemia and abdominal pain due to a thickened subepithelial collagen layer in the stomach, which is diagnosed histologically from a gastric biopsy. Unlike adults, it often presents with stomach-only involvement and less frequently with autoimmune conditions, though associations are increasing.^[1–22]^

To the best of our knowledge, this is the first time that the combination of collagenous gastritis, hyperplastic polyps, and intraepithelial lymphocytosis of the duodenum has been reported. This case report adheres to the CARE reporting guidelines for the presentation of a case study.^[23]^

## 2. Case report

### 2.1. Patient information

A 15-year-old male with a 2-year history of chronic abdominal pain, diarrhea, and iron deficiency anemia presented for evaluation. He also reported significant fatigue, intermittent constipation, and occasional bloating. Previous oral iron supplementation had been ineffective, necessitating the use of intravenous iron infusions. His symptoms had progressively worsened. His past medical history was notable for allergic rhinitis, with no prior history of gastrointestinal bleeding, weight loss, or fever. Family history included his father’s diagnosis of colon cancer at age 45. No autoimmune diseases were reported in the family. On examination, the patient was pale but otherwise appeared well. His body mass index was 16.02 kg/m², with a weight of 50 kg (25th percentile) and a height of 178 cm (88th percentile). Abdominal examination was unremarkable, with no tenderness, organomegaly, or distension. No peripheral stigmata of chronic disease were noted.

**Figure 1.**
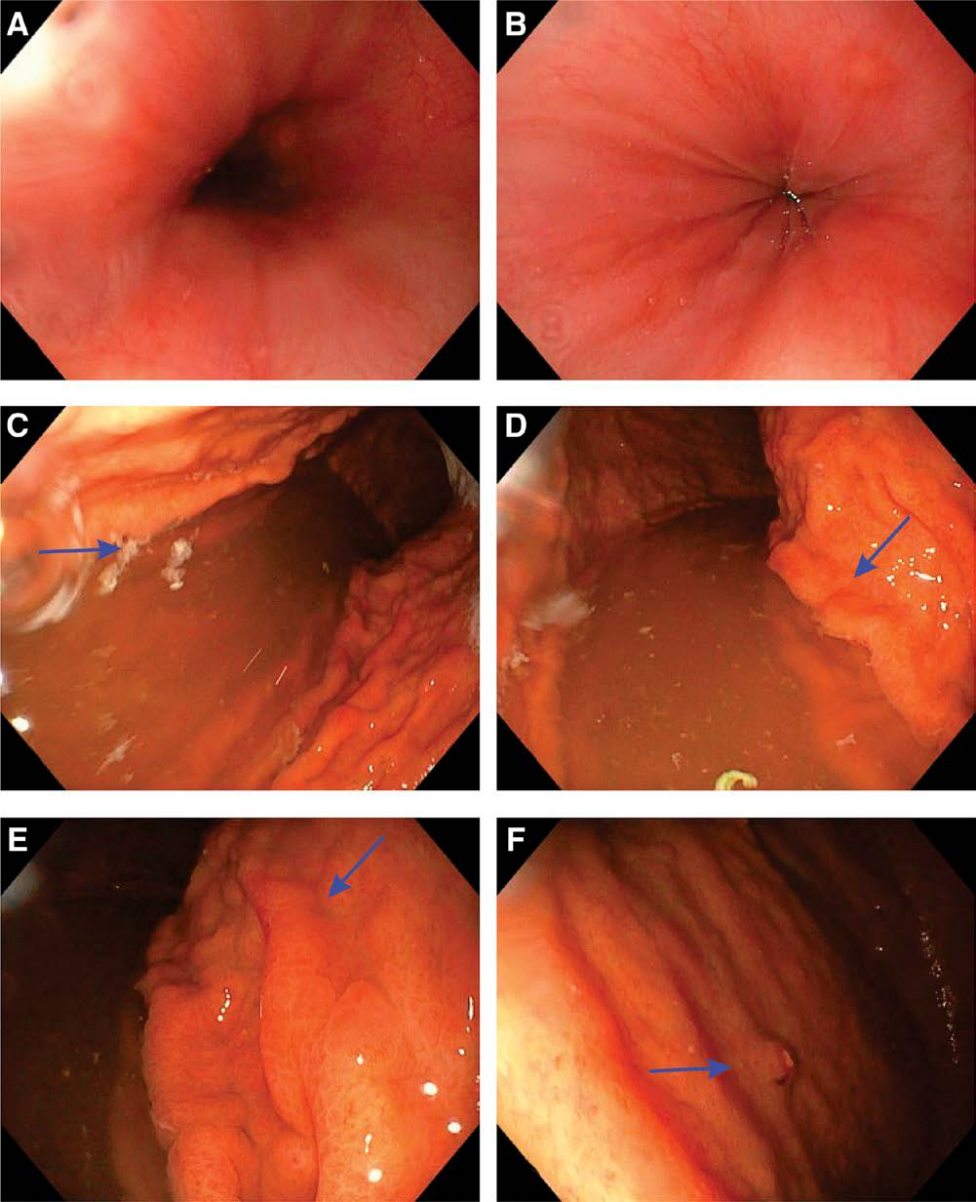
Endoscopy. (A, B) Esophagus with normal appearance. (C, D) Stomach with thickened mucosal folds. (E) Stomach with thickened mucosal folds and nodularity. (F) Stomach with a polypoidal lesion.

Initial laboratory investigations revealed a hemoglobin level of 130 g/L, a mean corpuscular volume of 72 fL, and a ferritin level of 9 µg/L. Before his assessment in the pediatric GI clinic, his lowest recorded hemoglobin level was 85 g/L, with a ferritin level of 3 µg/L, necessitating an IV iron infusion. Inflammatory markers, including C-reactive protein and erythrocyte sedimentation rate, were within normal limits. Fecal calprotectin was normal (74 µg/g). Celiac serology, including anti-tissue transglutaminase antibodies, was negative. Stool studies were negative for occult blood, parasites, and pathogens. Upper and lower gastrointestinal endoscopies were performed to further investigate the cause of anemia and chronic symptoms. The esophageal and colonic mucosa were grossly normal. However, the gastric mucosa exhibited marked nodularity, with thickened mucosal folds and small polypoid lesions, predominantly in the antral region (Fig. 1A–F). Biopsies were obtained from the esophagus, stomach, and colon. Gastric biopsies were consistent with a diagnosis of collagenous gastritis.

An ethics committee or institutional review board approval is waived according to the guidelines of the local hospital. The child’s parents or guardians signed an informed written consent form. Approval was waived once the informed written consent form was signed. The consent form has been uploaded to his electronic file in the Children’s Hospital of Eastern Ontario EPIC Electronic Medical Record, Ottawa, Ontario, Canada.

### 2.2. Diagnostic assessment

Microscopically, there was noticeable fibrosis of the lamina propria and upgrowth of smooth muscle from the muscularis mucosa in some areas of apparent atrophy. In certain regions, the surface epithelium was partially detached, and neutrophils had focally infiltrated the lamina propria as well. Below the surface epithelium or at the level of the foveolae in the mucosa, visible bands of hyalinized collagen were partly continuous. These bands contained entrapped capillaries, lymphocytes, and focal eosinophils. These collagen bands were blue when stained with Masson trichromic staining. The definitive diagnosis of collagenous gastritis was based on the biopsy that showed a subepithelial band of collagen in the mucosa that was thicker than 10 µm. Overlying these bands was epithelium that appeared normal in some areas and shorter and more disconnected in others. The number of white blood cells within the epithelium did not rise. *Helicobacter pylori* microorganisms were not detected with the hematoxylin and eosin staining and the modified Giemsa stain for bacteria. Moreover, the polypoid lesion demonstrated endoscopically in Figure 1F showed classic features of a hyperplastic polyp.

**Figure 2.**
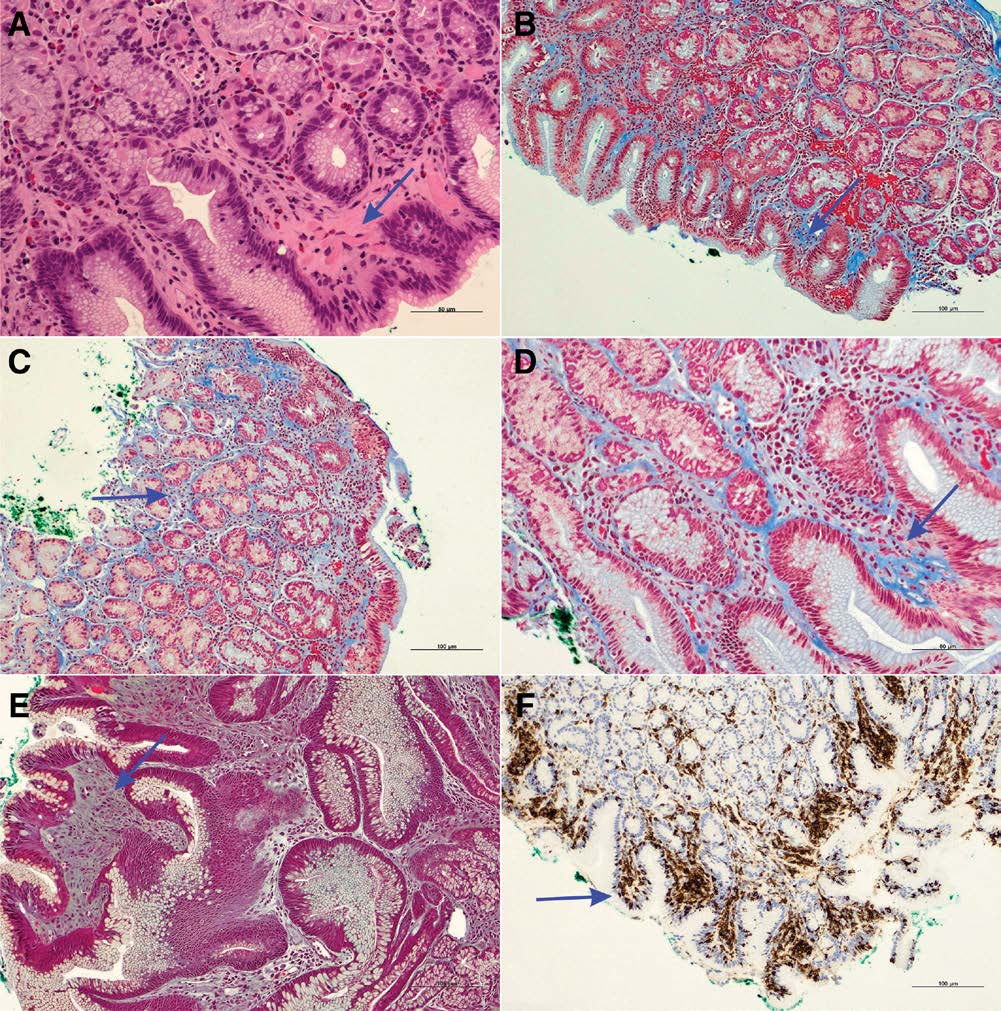
Histology, histochemistry, and immunohistochemistry. Gastric mucosa showing some scattered inflammatory cells with few eosinophils and a thick subepithelial band (A, arrow, scale bar embedded), which is highlighted by the blue staining using Masson trichromic (B–D, arrow, scale bars embedded). The collagen deposition percolated among the foveolae (B–D, scale bars embedded). The microphotograph in (E) demonstrates the histology of the hyperplastic polyp with dilated glands (Movat pentachrome staining, scale bar embedded). The microphotograph in (F) shows the CD3 immunohistochemistry of the duodenal epithelium, with mostly apical localization of the lymphocytes (scale bar embedded). There is no apparent villous blunting. Original magnifications: (A, D): 200×; (B, C, E, F): 100×.

The biopsy samples taken from the duodenum revealed minimal chronic inflammation with intraepithelial lymphocytosis exceeding 30 lymphocytes per 100 epithelial cells, which was confirmed using an antibody against the lymphocytic CD3 antigen. The lymphocytes were typically apically located in at least several villi. However, villous blunting was not present, which may correspond to Marsh I (Fig. 2).

**Figure 3.**
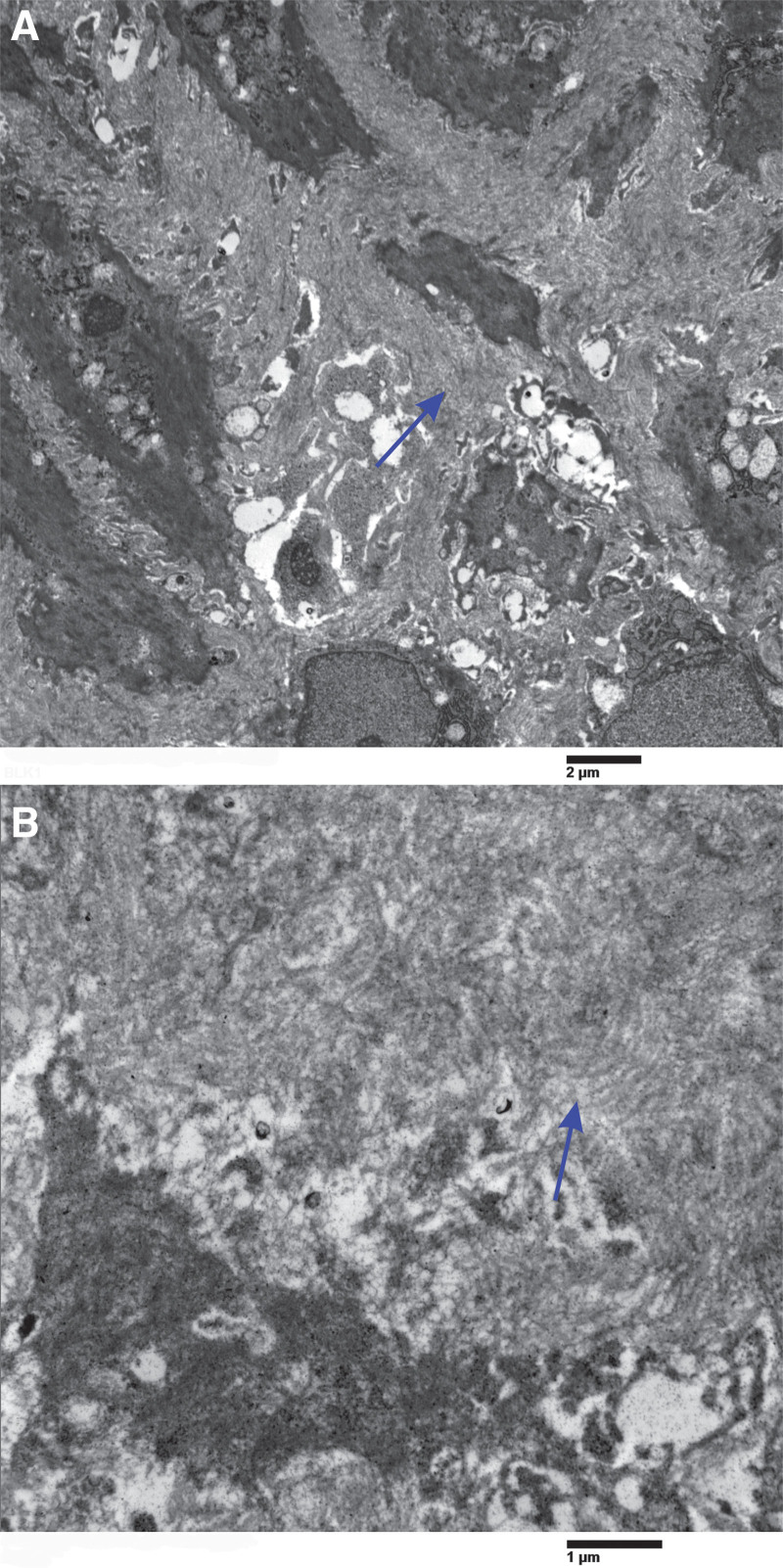
Electron microscopy. There is a marked increase in the thickness of the subepithelial band (A) and haphazardly distributed collagen fibers (B). Scale bar in (A): 1 μm, scale bar in (B): 2 μm. At places, an apparent reduplication of the basal membrane was noted. Original magnifications: (A) 5000×; (B) 15,000×.

On electron microscopy, gastric biopsy samples revealed a subepithelial band of collagen fibers that was ultrastructurally irregularly distributed (Fig. 3). Within this band, we could observe capillaries, lymphocytes, scattered eosinophils, mast cells, and fibroblasts. Surface epithelium separation from the membrane, enlargement of intercellular gaps, and localized reduction in mucous granules were also observed. There were no notable changes observed in fibroblasts situated near glands and foveolae that were ensnared in subepithelial collagen bundles. Some entrapped eosinophils, as well as those in the superficial and deep lamina propria, exhibited mild degranulation. The child was diagnosed with anemia because of continuous tiredness and low energy (hemoglobin level 85 g/L 1 year before the diagnosis). It was diagnosed as anemia (microcytic hypochromic anemia, mean corpuscular volume 68 when Hb 85, MCH 19) 1 year before the diagnosis of collagenous gastritis was rendered. Serum calprotectin and amyloid A levels were evaluated and were unremarkable or within the normal range.

### 2.3. Therapeutic intervention and outcome

**Figure 4.**
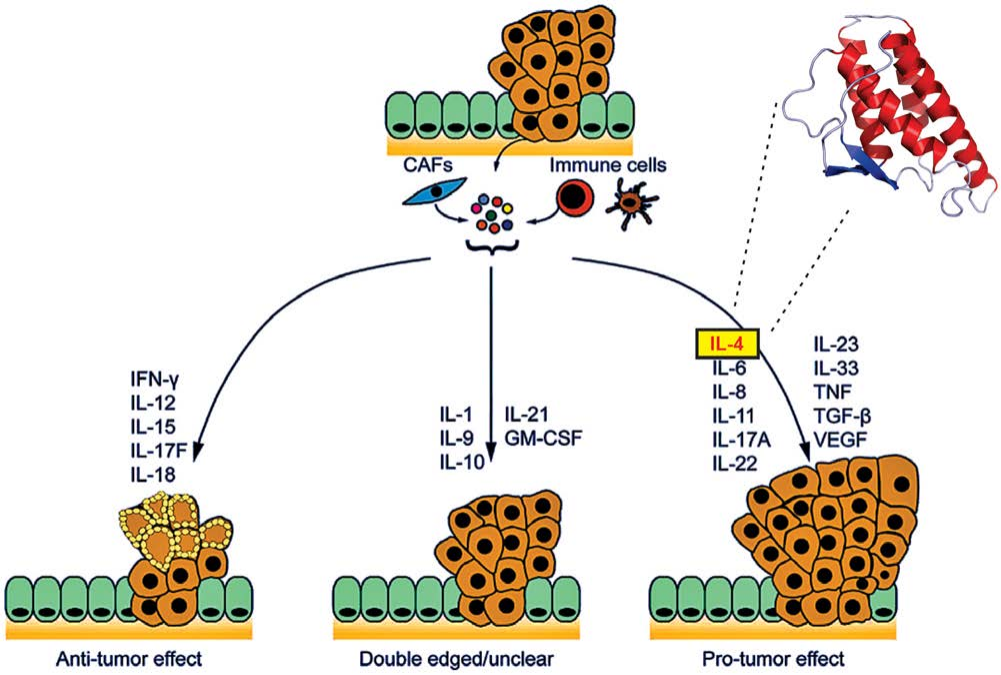
Interleukin (IL)-4's role in neoplastic progression. Cytokines produced by tumor and/or stromal cells aggregate to create networks with antitumor, protumor, or dual characteristics. Interferon (IFN)-γ, IL-12, IL-15, IL-17F, and IL-18 impair the progression of colorectal cancer. IL-4, IL-6, IL-8, IL-11, IL-17A, IL-22, IL-23, IL-33, TNF, TGF-β, and VEGF have protumorigenic properties. The roles of IL-1, IL-9, IL-10, IL-21, and GM-CSF in intestinal cancer are still unclear. Adapted from Mager et al.^[24]^

The child was started on lansoprazole 30 mg twice daily and famotidine 40 mg once daily, and oral iron supplementation was continued. He showed clinical improvement. More recent results showed a hemoglobin level of 123 g/L and a ferritin level of 9 µg/L while on iron supplementation. In consideration of a colon-rectal cancer diagnosis of his father at the age of 45 years, we carried out a Search Tool for the Retrieval of Interacting Genes/Proteins (STRING) and Pymol-based Protein Data Bank study and found that interleukin-4 (IL-4) may be the key factor supporting the occurrence of hyperplastic polyps in collagenous gastritis (Fig. 4). In molecular biology, STRING is a biological database and web resource of known and predicted protein–protein interactions. The STRING database comprises information from various sources, including experimental data, computational prediction methods, and publicly available text collections.

Currently, the status of the father is well and alive. He was treated successfully. No other medical issues or illnesses have been recorded in the family. The justification for a combination of PPI and H2 blockers is that the child was started on lansoprazole 30 mg twice daily and famotidine 40 mg once daily because the addition of the H2 blocker improved his symptoms. The options of vedolizumab infusions and topical budesonide were not considered because the patient improved with a combination of PPI and H2 blocker, as well as iron therapy. These options are planned if clinical symptomatology worsens.

## 3. Discussion

Collagenous gastritis is a rare disorder of the gastrointestinal tract characterized histologically by inflammation of the stomach’s mucosa, accompanied by increased subepithelial deposition of collagen. Little is known about the etiopathogenesis and natural history of collagenous gastritis, as the condition is relatively infrequent, particularly in childhood.^[1,25,26]^

This condition has been previously documented in 3 different ways: in isolation, with synchronous lymphocytic colitis, and with synchronous collagenous duodenitis and colitis.^[27]^ This case is unique in that it presents with collagenous gastritis, hyperplastic polyps, and non-celiac intraepithelial duodenal lymphocytosis.

Although stomach nodularity has been reported in some prior studies, our endoscopic findings confirmed extensive mucosal involvement. The pathophysiology of the subepithelial collagen band has been proposed to have 3 leading causes: inflammation, a localized anomaly of the pericryptal collagen sheath; and/or autoimmune damage.^[27]^ The histology and ultrastructural results in our situation appear to support the first 2 hypotheses. Occasionally, nonprogressive patchy atrophic gastritis is recognized.

Evidence from our study and previous studies suggests a shared or identical initial stimulus for atrophic gastritis and collagen band deposition; however, once initiated, subepithelial collagen deposition persists independently of gastritis progression in other areas of the mucosa. This would suggest that a local defect caused the specific deposition of collagen under the epithelium. Since the peri-epithelial fibroblastic sheath is the source of collagen in this zone, the defect would most likely be related to the malfunctioning of these cells. Collagenous gastritis and synchronous or metachronous occurrence of collagenous colitis in some patients could be explained by an inherent metabolic defect in these pericryptal fibroblasts, which is related to activated fibroblasts in the rectal mucosa.^[13,14,28,29]^

Regarding the addition of an H2 receptor antagonist (H2RA, H2 blockers) to a PPI, we found that instances of the combination of PPI and H2RA are not common and are occasionally used to control nocturnal acid spikes or breakthroughs. In fact, Mainie et al. found that the addition of a bedtime H2RA to PPI reduces the percentage of time with intragastric pH < 4 and nocturnal acid spikes/breakthroughs. H2RA should be considered as adjunct therapy in patients with persistent symptoms.^[30]^

Ultrastructural analysis of our patient’s gastric mucosa revealed some degranulating eosinophils in the lamina propria. In addition to being associated with certain fibrosing illnesses, eosinophils are known to cause tissue damage.^[18,19]^ While their activation may be the result of an inflammatory response that is not particular to any 1 disease, it is plausible that they contribute to the development of some collagenous gastrointestinal illnesses.

In a Swedish study of pediatric collagenous gastritis,^[1]^ the authors emphasize that childhood-onset collagenous gastritis seems to possess a chronic course exhibiting no endoscopic or histologic improvement. Moreover, these authors noted a high frequency of recurrent iron deficiency, as observed in our patient. The Swedish cohort commonly detected the inheritance for autoimmune disorders and autoantibodies, but it also occasionally identified the early development of autoimmune comorbidities. There was no apparent evidence of an association between childhood-onset collagenous gastritis and HLA DQ2/DQ8 haplotypes, which entail genes that are typically linked to celiac disease and, at least only rarely, to non-celiac wheat sensitivity.^[31,32]^ An ongoing follow-up study should be offered to all pediatric patients with collagenous gastritis, as they may eventually develop gluten-sensitive enteropathy.

The occurrence of collagenous gastritis and hyperplastic polyps was previously reported only in an abstract.^[33]^ In that report, the authors presented a 9-year-old girl with iron deficiency anemia and a nodular polypoid-appearing stomach with a single pedunculated polyp. A thickened subepithelial collagen band, associated with mixed chronic inflammation and atrophy, was observed in addition to a hyperplastic polyp. Focal intraepithelial lymphocytes were also seen in the small bowel.

IL-4 is a pleiotropic cytokine that regulates diverse T and B cell responses, including cell proliferation, survival, and gene expression. It has essential effects on the growth and differentiation of different immunologically competent cells.^[24]^ Liu et al.,^[34]^ using NanoString nCounter technology, identified that collagenous gastritis featured enhanced expression of key genes encoding both Th1 (IFNγ, TNF‐α, IL‐2, IL‐10, IL‐12A, IL‐12B, and IL‐18) and Th2 cytokines (IL‐3, IL‐4, IL‐5, IL‐6, and IL‐13). In contrast, biopsies from patients with collagenous colitis exhibited upregulated Th1 cytokines only. We conducted a STRING and PyMOL-based Protein Data Bank study and found that IL-4 may be a key factor supporting the occurrence of hyperplastic polyps in collagenous gastritis. A limitation of this study is that we are unable to measure IL-4 in serum or tissue; however, we can provide some thorough explanations that suggest IL-4 may play a key role in the neoplastic transformation. Family history included his father’s diagnosis of colon cancer at age 45.

IL-4 is overexpressed during the initial stages of colorectal cancer (CRC) development, such as in hyperplastic polyps, adenomas, and serrated adenomas. However, in adenocarcinomas, IL-4 levels do not show an increase relative to normal mucosa.^[35]^ Moreover, elevated serum levels of IL-4 were observed in CRC patients with distant metastases (M1) in comparison to those without metastases (M0). The presence of a Th2 gene signature, which includes IL-4, IL-5, and IL-13, in human colorectal cancer does not appear to have prognostic significance.^[24]^

IL-4 promotes the development of hyperplastic polyps within the inflammatory context of collagenous gastritis. The clinical implication is that IL-4 could be a biomarker for increased polyp formation in this condition and a potential therapeutic target.^[24]^ Blocking IL-4 activity might be a strategy to reduce or prevent the development of these polyps. Though the primary role of hyperplastic polyps is benign, some risk of malignant transformation exists with certain types of gastritis. IL-4, as a driver of this process, could be a focus for future diagnostic and therapeutic approaches.

In experimental animal models of colorectal cancer, IL-4-deficient mice treated with azoxymethane (AOM) showed a lower tumor incidence than wild-type mice.^[36]^ The AOM/dextran sodium sulfate (DSS) model is a widely used mouse model for studying colitis-associated CRC. It involves administering AOM, a chemical carcinogen, followed by repeated cycles of DSS, which induces colitis. This combination mimics key aspects of human CRC, including tumor location and type.^[37]^ In the AOM/DSS model of tumorigenesis, signaling through the IL-4 receptor α promoted intestinal tumor growth.^[38]^ A pro-tumorigenic function of IL-4 in colorectal cancer. Th2 cells and double-positive CD4+CD8αβ+αβ T cells, in conjunction with cancer-initiating cells, represent important sources of secreted IL-4 in CRC.^[39,40]^ The in vitro coculture of IL-4-secreting colorectal cancer-derived tumor-initiating cells with peripheral blood mononuclear cells resulted in the inhibition of peripheral blood mononuclear cell proliferation, which the introduction of IL-4-blocking antibodies could reverse. This mechanism may enable tumor-initiating cells to evade immune surveillance, thereby promoting CRC progression.^[40]^ The interaction between IL-4 and its receptor leads to the phosphorylation of signal transducer and activator of transcription-6 in hematopoietic and epithelial cells. In colorectal cancer tumors, increased phosphorylation of signal transducer and activator of transcription-6 is associated with poorer patient survival.^[41]^

## 4. Conclusion

We comprehensively report on the occurrence of a combination of collagenous gastritis, hyperplastic polyps, and duodenal intraepithelial lymphocytosis. This rare combination should alert both clinicians and pathologists to the importance of assessing autoimmune disorders, particularly childhood-onset collagenous gastritis, in the future. In consideration of a CRC diagnosis of his father at the age of 45 years, we carried out a STRING and Pymol-based Protein Data Bank study. We found that IL-4 may be the key factor supporting the occurrence of hyperplastic polyps in collagenous gastritis.

## Acknowledgments

We are grateful to the family. We are also grateful to all the researchers, including the physicians, nurses, and technicians, who participated in this study.

## Author contributions

**Conceptualization:** Consolato Sergi, Mohit Kehar.

**Data curation:** Consolato Sergi.

**Formal analysis:** Consolato Sergi, Janice Patry, Mohit Kehar.

**Funding acquisition:** Consolato Sergi.

**Investigation:** Consolato Sergi, Janice Patry, Mohit Kehar.

**Methodology:** Consolato Sergi.

**Project administration:** Consolato Sergi.

**Resources:** Consolato Sergi.

**Software:** Consolato Sergi.

**Supervision:** Consolato Sergi, Mohit Kehar.

**Validation:** Consolato Sergi.

**Visualization:** Consolato Sergi.

**Writing – original draft:** Consolato Sergi.

**Writing – review & editing:** Consolato Sergi, Janice Patry, Mohit Kehar.
